# PASNA syndrome with overlapping *CACNA1D* and *MED12L* variants: expanding the spectrum

**DOI:** 10.1210/jcemcr/luag131

**Published:** 2026-07-01

**Authors:** Bhanu Teja Nalluri, Neelaveni Kudugunti, Rakesh Kumar Sahay, Venkatesh Santhoju

**Affiliations:** Department of Endocrinology, Osmania Medical College, Hyderabad 500001, India; Department of Endocrinology, Osmania Medical College, Hyderabad 500001, India; Department of Endocrinology, Osmania Medical College, Hyderabad 500001, India; Department of Endocrinology, Osmania Medical College, Hyderabad 500001, India

**Keywords:** PASNA syndrome, *CACNA1D*, *MED12L*, infantile hypertension, developmental delay, hyperaldosteronism

## Abstract

Hypertension in infancy is rare and usually secondary to renal, cardiovascular, or endocrine disorders. PASNA syndrome (primary aldosteronism, seizures, and neurological abnormalities) (OMIM #615474) caused by activating *CACNA1D* mutations, has been increasingly recognized with wider availability of genetic testing. We report a 5-month-old male infant with incidentally detected severe hypertension (>99th percentile), neonatal jitteriness, and global developmental delay. Laboratory evaluation showed markedly elevated aldosterone (824 ng/dL) (SI: 22.9 nmol/L) (reference range, 5-150 ng/dL [SI: 0.14-4.2 nmol/L]) with low-normal renin and normal renal and cardiac imaging. Genetic analysis revealed a heterozygous *CACNA1D* missense variant (c.2241C > G; p.Phe747Leu) likely pathogenic and a heterozygous *MED12L* variant, classified as variants of unknown significance. The mother was an asymptomatic carrier of the *CACNA1D* variant. Blood pressure improved with nifedipine followed by spironolactone (2.5 mg/kg/day). Neurological symptoms partially improved, though motor delay persisted. This case expands the phenotypic spectrum of PASNA syndrome and highlights the need for early genetic evaluation in infants with unexplained hypertension and neurological abnormalities. The concurrent *MED12L* variant may contribute to the neurodevelopmental phenotype, underscoring the complexity of dual-gene involvement.

## Introduction

Hypertension in infancy poses a significant diagnostic challenge due to its rarity, diverse etiologies, and the necessity of distinguishing true hypertension from measurement artifacts. It is estimated to occur in less than 1% of neonates and infants, with diagnosis based on standardized blood pressure reference ranges adjusted for age, sex, and weight. When present, hypertension in this age group is almost always secondary, most commonly resulting from renal parenchymal or renovascular disease, followed by congenital heart defects (particularly coarctation of the aorta), endocrine abnormalities, and rare monogenic syndromes.

Advances in genomic sequencing over the past two decades have greatly enhanced the identification of monogenic forms of early onset hypertension. Among these, PASNA syndrome—characterized by primary aldosteronism, seizures, and neurological abnormalities—has been recognized as a distinct entity caused by activating mutations in the *CACNA1D* gene [1]. This gene encodes the Cav1.3 L-type calcium channel, which is expressed in the adrenal zona glomerulosa, pancreatic β-cells, and neuronal tissues [1]. Dysregulated channel activation results in excessive aldosterone secretion, autonomic imbalance, and neuronal dysfunction [2].

First described by Scholl et al in 2013, both somatic and germline *CACNA1D* activating mutations have been reported in patients with aldosterone-producing adenomas and syndromic neurodevelopmental features [1]. Subsequent reports have expanded the clinical spectrum to include autism spectrum disorders, intellectual disability, seizures, and variable degrees of hypertension [3-5]. Fewer than 20 cases have been documented worldwide, highlighting the rarity and phenotypic heterogeneity of this condition [2].

Here, we describe a rare case of PASNA syndrome in an infant who presented with incidentally detected hypertension and developmental delay. Genetic testing revealed a *CACNA1D* mutation along with a concurrent *MED12L* (variants of unknown significance). The latter, implicated in neurodevelopmental regulation, may be contributing to the broader neurological manifestations observed in our patient [6].

## Case presentation

A 5-month-old male infant was referred for evaluation of unexplained severe hypertension detected during a routine pediatric visit. He was the second child of non-consanguineous parents. The mother, aged 31 years, was a known case of hypothyroidism on regular medication, with no history of gestational hypertension or diabetes. The father, aged 32 years, was healthy with no comorbidities, and there was no significant family history of hypertension or renal disease. The couple's first child was healthy with normal growth and development. The antenatal period of the current pregnancy was uneventful, with normal fetal growth and anatomy on routine scans. The infant was delivered at 38 weeks of gestation via elective lower segment cesarean section with a birth weight of 2.8 kg, length of 48 cm, and head circumference of 34 cm. He cried immediately after birth and required no resuscitation.

### Neonatal course

On day 3 of life, the infant developed recurrent episodes of jitteriness and brief tonic stiffening, raising concern for neonatal seizures. However, blood glucose, calcium, magnesium, and other electrolytes were normal. There was no history of hypoxic insult or sepsis. An EEG (electroencephalogram) performed at the time showed no epileptiform activity, and the episodes were diagnosed as benign neonatal jitteriness. The infant was discharged without anticonvulsant therapy.

### Developmental history

During early infancy, the caregivers observed persistent irritability, poor feeding, and abnormal movements. Developmental delays became apparent—the child failed to achieve a social smile by 2.5 months, had inadequate head control by 4 months, showed poor visual engagement, minimal environmental interest, and reduced auditory responsiveness. Parents also reported reduced spontaneous limb movements and stiffness on handling.

### Clinical examination at 5 months

Anthropometric assessment showed that the infant weighed 6.1 kg, which is below the 10th percentile, measured 63 cm in length (between the 10th and 25th percentiles), and had a head circumference of 41.8 cm, also below the 10th percentile. The infant was alert but irritable during the examination. Vital signs revealed a blood pressure of 120/70 mmHg, which is above the 99th percentile for age and was confirmed in all four limbs using an appropriately sized cuff. The heart rate was 142 beats per minute, the respiratory rate was 38 breaths per minute, and the oxygen saturation was 98% on room air. On general examination, there was mild periorbital puffiness, but the facies were otherwise normal, with no dysmorphic features or peripheral edema. Neurological examination demonstrated axial hypotonia with hypertonia of the limbs, brisk deep tendon reflexes, a weak palmar grasp, absent social smile, poor head control, and limited vocalization. No abnormal involuntary movements were observed. Cardiovascular examination showed normal heart sounds, no murmurs, and symmetrical peripheral pulses. The abdominal examination revealed no hepatosplenomegaly, no renal masses, and no ascites. Ophthalmologic evaluation showed normal optic discs with no evidence of papilledema or retinopathy. A hearing assessment could not be completed due to poor cooperation.

## Diagnostic assessment

Hematological evaluation revealed a hemoglobin level of 12.6 g/dL (SI: 126 g/L) (reference range, 11-14 g/dL [SI: 110-140 g/L]), with normal white blood cell and platelet counts. Renal function was preserved, with a serum creatinine of 0.25 mg/dL (SI: 22 µmol/L) (reference range, 0.16-0.31 mg/dL [SI: 18-35 µmol/L]) and blood urea of 8 mg/dL (SI: 2.9 mmol/L) (reference range, 7.0-18.2 mg/dL [SI: 2.5-6.5 mmol/L]). Serum electrolytes were within normal limits, including sodium of 138 mmol/L (reference range, 135-145 mmol/L) and potassium of 4.0 mmol/L (reference range, 3.5-5.0 mmol/L). Arterial blood gas analysis showed a pH of 7.36 (reference range, 7.35-7.45) and a bicarbonate (HCO_3_^−^) level of 18 mmol/L (reference range, 22-26 mmol/L), indicating a mild metabolic acidosis. The endocrine profile demonstrated a plasma renin activity of 4.2 ng/mL/hour (reference range, 2-35 ng/mL/hour) and a markedly elevated serum aldosterone of 824 ng/dL (SI: 22.9 nmol/L). Urinalysis showed no proteinuria, hematuria, or abnormal casts. Liver and thyroid function tests were within normal limits. A metabolic screen using tandem mass spectrometry was negative for inborn errors of metabolism.

Imaging studies included an abdominal ultrasound showing normal kidneys, adrenal glands, and urinary bladder, and a renal artery doppler that demonstrated no evidence of renovascular abnormalities. Echocardiography revealed a structurally normal heart without left ventricular hypertrophy or coarctation of the aorta. Cranial ultrasound showed normal ventricular size and parenchymal echotexture, and an EEG demonstrated age-appropriate background activity with no epileptiform discharges.

Genetic analysis: Whole exon sequencing for monogenic hypertension and neurodevelopmental disorders revealed:

1. *CACNA1D* missense variant (c.2241C > G; p.Phe747Leu) (NM_000720.3): heterozygous, autosomal dominant, likely pathogenic consistent with PASNA syndrome [1, 3-5, 7].
2. *MED12L* missense variant (c.4364G > C; p.Arg1455Thr) (NM_ 053002.5): heterozygous, autosomal dominant, variant of unknown significance, associated with NIZON-ISIDOR syndrome [6].

Parental segregation analysis revealed that the mother is heterozygous for the same *CACNA1D* variant, while the father tested negative; the mother and father were negative for the *MED12L* variant. Despite carrying the *CACNA1D* variant, the mother is clinically asymptomatic. Given the autosomal dominant inheritance pattern of PASNA, this finding is consistent with reduced or variable penetrance. According to ACMG/AMP (American College of Medical Genetics and Genomics and the Association for Molecular Pathology) guidelines, the presence of the variant in an unaffected parent limits the strength of segregation evidence (PP1) but does not preclude pathogenicity. Previously published functional studies demonstrating gain-of-function effects support application of functional evidence (PS3); however, final classification integrates functional data, segregation analysis, population frequency, and phenotype specificity within the ACMG framework [4, 8].

Differential diagnoses considered included renal hypertension, which was excluded based on normal renal function, doppler findings, and urinalysis. Adrenal tumor (aldosterone-producing adenoma) was ruled out by normal abdominal imaging without adrenal masses. Coarctation of the aorta was excluded with normal echocardiogram and symmetrical limb blood pressures. Congenital adrenal hyperplasia was unlikely given normal electrolytes, absence of virilization or genital anomalies, and normal 17-hydroxyprogesterone. Other monogenic mineralocorticoid-excess syndromes (eg, apparent mineralocorticoid excess, gordon syndrome) was ruled out by biochemical profile and confirmed *CACNA1D* mutation identifying PASNA as the etiology [1, 4, 5].

## Treatment

The infant was initially started on sustained release nifedipine (0.5 mg/kg/day, titrated up to 2 mg/kg/day), which achieved partial blood pressure reduction. Following confirmation of PASNA syndrome, therapy was transitioned to spironolactone (2.5 mg/kg/day), resulting in normalization of blood pressure without electrolyte disturbances or hypotension [1, 4-6]. Neurology and physiotherapy consultations were initiated to address tone abnormalities and developmental delay. Genetic counseling was provided to the parents, focusing on the inheritance pattern, incomplete penetrance, and reproductive implications.

## Outcome and follow-up

At the 3- and 6-month follow-up visits, the infant's blood pressure remained well controlled on spironolactone monotherapy. There were no further episodes of jitteriness or seizure-like events. By 10 months of age, motor tone had partially improved with physiotherapy; however, global developmental delay persisted, particularly in the motor and social domains. Feeding difficulties continued, and growth parameters remained below the third percentile.

This comprehensive evaluation confirmed PASNA syndrome due to a *CACNA1D* mutation, with an additional *MED12L* variant (variant of unknown significance) may be contributing to the neurodevelopmental phenotype.

## Discussion

Voltage-gated calcium channels (VGCCs) play a pivotal role in coupling membrane depolarization to calcium influx, thereby regulating hormone secretion, neuronal excitability, and muscle contraction. The *CACNA1D* gene encodes the α1-subunit of the Cav1.3 L-type calcium channel, which is expressed in adrenal zona glomerulosa and in neurons. Gain-of-function mutations in *CACNA1D* lead to increased calcium entry at lower depolarization thresholds, resulting in excessive aldosterone synthesis and altered neuronal activity. This forms the pathophysiologic basis of the recently recognized syndrome of PASNA [1, 4-6, 9].

The association between *CACNA1D* mutations and aldosterone excess was first demonstrated by Scholl et al (2013), who identified somatic and germline variants (notably p.Gly403Asp (NM_001128840.2 (https://www.ncbi.nlm.nih.gov/nuccore/NM_001128840.2):c.1207G>C) and p.Ile770Met (NM_000720.3 (https://www.ncbi.nlm.nih.gov/nuccore/NM_000720.3):c.2310C>G; and in different isoform NM_001128840.2 (https://www.ncbi.nlm.nih.gov/nuccore/NM_001128840.2):c.2250C>G)) in patients with early onset severe hypertension and elevated aldosterone with suppressed renin [1]. These gain-of-function mutations shifted channel activation to more hyperpolarized potentials, leading to calcium-dependent overproduction of aldosterone. Affected infants also exhibited seizures and global developmental delay, establishing the concept of a syndromic form of hyperaldosteronism.

Subsequently, Pinggera et al (2015) expanded the clinical and molecular spectrum by describing de novo *CACNA1D* mutations (including p. Ala749Gly) (NM_000720 (https://www.ncbi.nlm.nih.gov/nuccore/NM_000720) reference sequence) in children with autism spectrum disorder, seizures, and variable hypertension [3]. Functional characterization confirmed enhanced Cav1.3 activity, linking these variants to both endocrine and neurodevelopmental phenotypes. The neurological manifestations were often more disabling than the hypertensive component, highlighting the dual adrenal–neuronal consequences of Cav1.3 channel overactivity.

More recently, Török et al (2023) reported a neonatal case with a de novo p.Phe767Ser mutation (reference sequence: NM_000720.3 [https://www.ncbi.nlm.nih.gov/nuccore/NM_000720.3]) presenting in the first week of life with severe hypertension, jitteriness, transient hypoglycemia, and developmental delay [4]. This mutation, located in the S6 transmembrane segment of domain II—a critical gating region—produced marked gain-of-function effects similar to earlier variants. The clustering of pathogenic residues around positions 740-770 (including Phe747, Ala749, and Ile770) underscores the functional hotspot responsible for excessive calcium channel activity.

Collectively as depicted in Table 1, these reports establish *CACNA1D* as a cause of early onset, often neonatal, hypertension associated with elevated aldosterone and neurodevelopmental impairment. Despite effective blood pressure control with calcium-channel blockers or mineralocorticoid receptor antagonists, neurological deficits frequently persist [1, 4-6, 9]. Early genetic testing in infants with unexplained hypertension and developmental abnormalities is therefore essential for diagnosis, prognostication, and exploration of targeted Cav1.3-specific therapies.

**Table 1:** Table describing the review of cases of PASNA syndrome with presentation, neurological features, and management

Author/Year	Age at presentation with hypertension	CACNA1D mutation	Hypertension	Neurological features	Hyperinsulinism with hypoglycemia	Aldosterone/Renin status	Treatment given	Outcome
Scholl et al, 2013 [1]	Neonatal period	p.Gly403Asp (c.1207G > C) (NM_001128840.2 (https://www.ncbi.nlm.nih.gov/nuccore/NM_001128840.2))	Severe (>99th percentile)	Seizures, cerebral palsy-like features, developmental delay	No	↑ Aldosterone/↓ Renin (suppressed)	Spironolactone, Amlodipine	BP control; persistent developmental delay
Scholl et al, 2013 [1]	5 years	p.Ile770Met (c.2310C > G) (NM_000720.3 (https://www.ncbi.nlm.nih.gov/nuccore/NM_000720.3) and in different isoform NM_001128840.2 (https://www.ncbi.nlm.nih.gov/nuccore/NM_001128840.2):c.2250C>G)	Severe (>99th percentile)	Developmental delay, hypertonia Seizures	No	↑ Aldosterone/↓ Renin (suppressed)	Initially managed with clonidine followed by Spironolactone	BP improved; neurodevelopmental delay continues
Török et al, 2023 [4]	Neonatal period (4 weeks)	p.Phe767Ser (c.2300T > C) [F747S] (NM_000720.3 (https://www.ncbi.nlm.nih.gov/nuccore/NM_000720.3))	Severe (>99th percentile)	Jitteriness, transient hypoglycemia, developmental delay, hypotonia	Yes	↑ Aldosterone/↓ Renin (suppressed)	Nifedipine, Spironolactone	BP well-controlled; jitteriness resolved; transient hypoglycemia corrected; persistent motor delay on follow-up
De Mingo Alemany et al, 2020 [5]	Neonatal period	p.Leu271His (c.812 T > A) (NM_000720.3 (https://www.ncbi.nlm.nih.gov/nuccore/NM_000720.3))	Severe (>99th percentile)	Hyperreactivity, tremulations and hypotonia	Yes	↑ Aldosterone/↓ Renin (suppressed	Nifedipine and Diazoxide	BP improved; neurodevelopmental delay continues
Present Case, 2025	Neonatal period (<7 days)	p.Phe747leu (c.2241C > G) (NM_000720.3 (https://www.ncbi.nlm.nih.gov/nuccore/NM_000720.3))	Severe (120/70 mmHg, >99th percentile)	Jitteriness, hypotonia, delayed motor milestones, absent social smile, developmental delay	No	↑ Aldosterone (824 ng/dL) (SI: 22.9 nmol/L)*/Normal Renin (4.2 ng/mL/hr)*	Nifedipine (initial) → Spironolactone (2.5 mg/kg/day)	BP well-controlled; jitteriness improved; persistent motor delay with hypertonia; mild improvement on physiotherapy

Reference values.

Plasma renin activity (reference range, 2-35 ng/mL/hour).

Aldosterone—(reference range, 5-150 ng/dL [SI: 0.14-4.2 nmol/L]).

Variants in *MED12L* are classically associated with neurodevelopmental disorders characterized by intellectual disability, hypotonia, craniofacial dysmorphism, and corpus callosum abnormalities. However, the identified *MED12L* variant in this case lacks previously reported pathogenic features and does not correlate strongly with the patient's predominant PASNA phenotype. In silico prediction tools and available databases do not support a clearly deleterious effect, and there is no established genotype–phenotype overlap with classic PASNA syndrome. Therefore, the *MED12L* variant is unlikely to be pathogenic and may represent a variant of uncertain significance with only a minimal contributory role in this case of PASNA.

PASNA syndrome should be suspected in infants presenting with unexplained hypertension and neurological abnormalities [1, 2]. This case adds to the limited global literature by identifying a *CACNA1D* mutation coexisting with a *MED12L* variant, expanding the known neurological phenotype. Early genetic diagnosis facilitates targeted treatment, optimal blood pressure control, and informed family counseling. Future longitudinal studies and case registries are needed to refine prognostic predictors and therapeutic strategies in PASNA and related calcium channelopathies.

## Learning points

- Infantile hypertension is uncommon and typically secondary; monogenic etiologies such as PASNA syndrome should be suspected in unexplained cases [1, 2].
- PASNA is caused by gain-of-function *CACNA1D* mutations, resulting in aldosterone excess, hypertension, and neurological abnormalities [1, 3-5].
- Genetic testing is essential when routine evaluations do not reveal an underlying cause.
- Additional neurodevelopmental gene variants (*MED12L*) identified on exome sequencing may potentially broaden the neurological phenotype; however, their pathogenic significance should be interpreted with caution, as co-occurring variants may represent incidental or secondary findings rather than direct contributors to disease severity [6].
- Mineralocorticoid receptor antagonists like spironolactone effectively control hypertension, but neurodevelopmental outcomes are variable, underscoring the need for genetic counseling and family screening [1, 4, 5].

## Contributors

All authors made individual contributions to authorship. B.T.N., N.K., R.K.S., and V.S. were involved in the diagnosis and management of the patient. All authors edited, reviewed, and approved the final draft.

## Data Availability

Data sharing is not applicable to this article as no datasets were generated or analyzed during the present study.
